# Modeling the active dispersal of juvenile leatherback turtles in the North Atlantic Ocean

**DOI:** 10.1186/s40462-019-0149-5

**Published:** 2019-02-28

**Authors:** Maxime Lalire, Philippe Gaspar

**Affiliations:** Sustainable Management of Marine Ressources, Collecte Localisation Satellites, Ramonville Saint-Agne, France

**Keywords:** Leatherback sea turtle, Pelagic juvenile stage, Active dispersal, Individual Based Model, North Atlantic Ocean

## Abstract

**Background:**

The Northwest Atlantic (NWA) leatherback turtle (*Dermochelys coriacea*) subpopulation is one of the last healthy ones on Earth. Its conservation is thus of major importance for the conservation of the species itself. While adults are relatively well monitored, pelagic juveniles remain largely unobserved. In an attempt to reduce this knowledge gap, this paper presents the first detailed simulation of the open ocean dispersal of juveniles born on the main nesting beaches of French Guiana and Suriname (FGS).

**Methods:**

Dispersal is simulated using STAMM, an Individual Based Model in which juveniles actively disperse under the combined effects of oceanic currents and habitat-driven movements. For comparison purposes, passive dispersal under the sole effect of oceanic currents is also simulated.

**Results:**

Simulation results show that oceanic currents lead juveniles to cross the Atlantic at mid-latitudes. Unlike passive individuals, active juveniles undertake important north-south seasonal migrations while crossing the North Atlantic. They finally reach the European or North African coast and enter the Mediterranean Sea. Less than 4-year-old active turtles first arrive off Mauritania. Other productive areas on the eastern side of the Atlantic (the coast of Galicia and Portugal, the Gulf of Cadiz, the Bay of Biscay) and in the Mediterranean Sea are first reached by 6 to 9-year-old individuals. This active dispersal scheme, and its timing, appear to be consistent with all available stranding and bycatch data gathered on the Atlantic and Mediterranean coasts of Europe and North Africa. Simulation results also suggest that the timing of the dispersal and the quality of the habitats encountered by juveniles can, at least partly, explain why the NWA leatherback subpopulation is doing much better than the West Pacific one.

**Conclusion:**

This paper provides the first detailed simulation of the spatial and temporal distribution of juvenile leatherback turtles dispersing from their FGS nesting beaches into the North Atlantic Ocean and Mediterranean Sea. Simulation results, corroborated by stranding and bycatch data, pinpoint several important developmental areas on the eastern side of the Atlantic Ocean and in the Mediterranean Sea. These results shall help focus observation and conservation efforts in these critical areas.

**Electronic supplementary material:**

The online version of this article (10.1186/s40462-019-0149-5) contains supplementary material, which is available to authorized users.

## Background

The Northwest Atlantic (NWA) hosts one of the last healthy leatherback turtle (*Dermochelys coriacea*) subpopulations worldwide. With about 50,000 nests in 2010, and likely growing, this subpopulation is of major importance for the conservation of the species [1]. It comprises several nesting aggregations extending from French Guiana to Florida, including the whole mainland and insular Caribbean area. French Guiana and Suriname (FGS) together host nearly 50% of the nesting activity. This subpopulation is particularly well observed, at least at the adult stage.

The main FGS nesting beaches have been monitored for over a decade and sometimes over 30 years, thereby providing a solid basis for demographic studies [2, 3]. Although natal homing might be less accurate than in other sea turtle species, leatherbacks clearly display natal philopatry [4, 5]. Most FGS females come back, every two to three years [3], to nest on their natal beach. They then undertake, often long, post-nesting migrations towards favorable foraging grounds. Abundant satellite-tracking data reveal that they occupy productive, but often cold, areas during summer and fall and move toward somewhat warmer waters during winter [6–11]. Targeted foraging grounds are widely spread in the North Atlantic basin. The cold, but very favorable foraging area off Nova Scotia is highly used [12]. Other important foraging grounds are also exploited in the central and western Atlantic Ocean: around the Azores archipelago, along the European coast (specially off Ireland, the Bay of Biscay and Portugal) and along the north African coast (specially off Mauritania and around Cape Verde Islands). Adult males have been much less tracked but the few existing data suggest that males occupy the same foraging grounds as females and migrate, possibly every year, to be present near the nesting beaches early in the nesting season (spring). They stay there for a couple months, and likely breed, before migrating back towards their foraging grounds [13].

Unlike adults, juvenile NWA leatherback turtles have never been satellite-tracked and their spatial ecology remains largely unknown. This hampers the development of conservation measures focused on this critical life stage [14, 15]. This lack of information concerning the oceanic juvenile stage, often called “the lost years”, is common to most sea turtle populations. The main piece of information available on this life stage is that the sizes and spatial distribution of the few juveniles captured at sea or found stranded are consistent with the hypothesis that dispersal is largely governed by oceanic currents downstream of the nesting beaches [16, 17]. Based on such observations, it has commonly been assumed that juveniles drift purely passively with oceanic currents. Accordingly, the initial dispersal of various juvenile sea turtle populations has been investigated using simple Individual Based Models (IBM) in which trajectories of thousands of particles, each representing an individual, are simulated using freely available Lagrangian particle-tracking software forced by surface currents produced by ocean circulation models [18–23].

While oceanic currents might indeed be the main cause of dispersal during the first months of life of hatchlings and juveniles, evidence is mounting that juveniles do not drift purely passively but also swim actively [24, 25], often towards favorable habitats [24, 26, 27]. Surprisingly enough, the first IBMs simulating the impact of active swimming on juveniles’ dispersal did not focus on habitat-driven movements but rather attempted to reproduce the effects of occasional movements like the initial swimming frenzy [28] or oriented movements prompted by specific values of the Earth magnetic field [29, 30]. STAMM (Sea Turtle Active Movement Model), the first IBM simulating the dispersal of juvenile sea turtles under the combined effects of oceanic currents and habitat-driven swimming movements was only recently published Gaspar and Lalire [31], hereafter referred to as GL. It simulates swimming movements triggered by the need to find food and suitable water temperatures. STAMM was first used to simulate the dispersal of juvenile West Pacific leatherbacks into the North Pacific Ocean. The results of this first simulation demonstrate that the active dispersal scenario produced by STAMM is in better agreement with the few juvenile bycatch data available (off California and Hawaii) than the purely passive dispersal scenario [31].

Except for one short passive drift simulation [32], numerical models have never been used to investigate the dispersal of NWA juvenile leatherbacks. In an attempt to shed light on the lost years of this population, we present and analyse here the first numerical simulations of the long-term dispersal of NWA leatherback hatchlings emerging from the FGS nesting beaches. These simulations are performed using STAMM. As bycatch and stranding data are more numerous and better distributed in the North Atlantic than in the North Pacific Ocean, the simulations performed here offer an excellent opportunity to further validate STAMM. In addition, comparison of our simulations with these of GL will allow us to evaluate if the habitats encountered by the juveniles in the Atlantic are more favorable than in the Pacific and could therefore be a part of the explanation why the NWA leatherback subpopulation is doing better than the West Pacific one.

## Methods

### STAMM

STAMM is a generic IBM simulating the dispersal of juvenile sea turtles under the combined effects of oceanic currents and habitat-driven swimming motions. It was fully described by GL. We will only recall here the basic equations governing simulated movements and habitats. We will then calibrate them for juvenile leatherbacks dispersing in the NWA.

#### Movement model

Individuals, as simulated by STAMM, move with a velocity on the ground (***V***_***g***_) resulting from the current velocity (***V***_***c***_) and their own swimming velocity (***V***_***s***_):

1 $$ {\boldsymbol{V}}_{\boldsymbol{g}}={\boldsymbol{V}}_{\boldsymbol{c}}+{\boldsymbol{V}}_{\boldsymbol{s}} $$

Estimates of the current velocity can be obtained from any ocean circulation model. The swimming velocity of an individual of age *a*, at time *t* and position (*x*, *y*) is estimated using the model of Faugeras and Maury [33]:

2 $$ {\boldsymbol{V}}_{\boldsymbol{s}}\left(x,y,t,a\right)={V}_m(a)\left(1-h\right)\ \boldsymbol{d} $$

where *V*_*m*_ is the age-dependent maximum sustainable speed, *h* is a normalized habitat suitability index (0≤ *h* ≤1) and ***d*** is the unit vector pointing in the direction of movement:

3 $$ \boldsymbol{d}=\left(\sin \theta, \cos \theta \right) $$

with *θ* the heading angle (relative to North).

The factor (1 − *h*) in Eq. (2) guarantees that the swimming speed is a monotonically decreasing function of habitat suitability so that individuals move rapidly through poor habitats and slowdown in favorable areas. The heading angle *θ* is taken to be a realization of a stochastic variable having a von Mises distribution *vM*(μ, κ) with mean direction angle μ and concentration parameter κ. The mean direction of movement is chosen to be the direction of the habitat gradient vector **∇*h***:

4 $$ \mu ={\theta}_{\mathbf{\nabla}\boldsymbol{h}} $$

and the concentration parameter κ is taken to be proportional to the norm of **∇*h***:

5 $$ \upkappa =\upalpha \left\Vert \mathbf{\nabla}\boldsymbol{h}\right\Vert $$

This parameterization of μ and κ guarantees that the movement direction is, on average, that of the habitat gradient (*θ*_**∇*h***_) and hence leads individuals towards more suitable habitats. It also guarantees that movements become increasingly directed towards favorable habitats as the habitat gradient (and hence the concentration parameter κ) increases. They become less directed as the habitat gradient decreases and get close to a random walk as ‖**∇*h***‖ → 0.

The maximum sustainable speed *V*_*m*_ is, by definition, the speed for which the amount of energy required to move one unit distance is minimum [34]. Noting *M* the mass of an individual, *L* its size, and assuming that the resting metabolic rate (*RMR*) scales with *M*^*b*^ while *M* scales with *L*^*c*^, a simple energy budget yields [31]:

6 $$ {V}_m={v}_0\ {L}^{\frac{bc-2}{3}} $$

where *v*_0_ is a species-dependent parameter. Given a growth curve *L*(a), this equation governs the evolution of *V*_*m*_ with age.

#### Habitat model

As simulated movements are governed by the need to find food and suitable water temperatures, the habitat suitability index (*h*) is expressed as the product of a thermal habitat index (*h*_*T*_) and a feeding habitat index (*h*_*F*_), both ranging between 0 and 1:

7 $$ h={h}_T{h}_F $$

The thermal habitat suitability index has to reflect the fact that, like all ectotherms, sea turtles can only perform in a limited range of body temperatures (*T*_*b*_). Given the high thermal conductivity of sea water, *T*_*b*_ is closely tied to the water temperature (*T*_*w*_). Sea turtles must thus remain in a limited range of *T*_*w*_ to avoid cold stunning or overheating. Such a bounded thermal habitat is modelled in STAMM using 4 pivotal temperatures *T*_*1*_ < *T*_*2*_ < *T*_*3*_ < *T*_*4*_. It reads:

8 $$ {\displaystyle \begin{array}{c}{h}_T\left(x,y,t,a\right)={\mathrm{e}}^{-2\ {\left(\frac{T_w-{T}_2}{T_2-{T}_1}\right)}^2}\kern0.5em if\kern0.5em {T}_w<{T}_2\\ {}=1\kern0.5em if\kern0.5em {T}_2\le {T}_w\le {T}_3\\ {}={\mathrm{e}}^{-2\ {\left(\frac{T_w-{T}_3}{T_4-{T}_3}\right)}^2}\kern0.5em if\kern0.5em {T}_w>{T}_3\end{array}} $$

*T*_*1*_ and *T*_*4*_ are critical temperatures below or above which an individual cannot survive for long (i.e. *h*_*T*_ ≈ 0 for *T*_*w*_ < *T*_*1*_ or *T*_*w*_ > *T*_*4*_) while *T*_*2*_ and *T*_*3*_ are the lower and upper bounds of the thermal preferendum, that is the minimum and maximum water temperatures between which a sea turtle performs optimally or nearly so (i.e. *h*_*T*_ = 1 for *T*_*2*_ ≤ *T*_*w*_ ≤ *T*_*3*_). These temperatures vary with the species and the size or mass of the individual.

The feeding habitat suitability index is simply taken to be proportional to *P*(*x*, *y*, *t*) the local prey density (or a proxy of it), divided by the individual rate of food consumption which evolves with age:

9 $$ {h}_F\left(x,y,t,a\right)=\mathit{\operatorname{Min}}\ \left[1,\frac{P\left(x,y,t\right)}{P_0{F}_0(a)}\right] $$

Where *P*_0_ is a scaling parameter and *F*_0_(*a*) is the rate of food consumption, normalized so that *F*_0_(*a*)→1 when *a* → + ∞. In practice, satellite-derived values of the net primary production (NPP) is used as a proxy for the prey density *P*.

### The leatherback version of STAMM

The above-described generic version of STAMM includes 7 parameters: 2 for the movement model (α and *v*_0_), 4 for the thermal habitat (*T*_*1*_ to *T*_*4*_) and one for the feeding habitat (*P*_0_). Five of them (*v*_0_, *T*_*1*_, *T*_*2*_, *T*_*3*_, *T*_*4*_) only depend on the modelled species. GL estimated them for leatherback turtles. We simply use the same estimates here (see Table 1). The velocity scaling parameter *v*_0_ is simply chosen so that, in average habitat conditions (*h* =0.5), the speed of adults is close to 0.6 m/s, a typical value observed in tracked leatherbacks. The two lower pivotal temperatures are derived from an individual steady-state heat budget based on which *T*_*1*_ and *T*_*2*_ are shown to decrease with *M*^0.5^ [31, 35]. This parameterizes the specific thermoregulatory ability of leatherbacks, also called gigantothermy [36], that allows individuals to venture into progressively colder waters as they grow [37]. Upper pivotal temperatures (*T*_*3*_ and *T*_*4*_) likely take high values, near 40 °C, the critical thermal maximum observed in hatchlings [38], and likely close to that of adults [39]. As seawater temperatures rarely exceed 30 °C in the open ocean, the condition *T*_*w*_ > *T*_*3*_ is, in practice, never met in Eq. (8). Therefore, the two parameters *T*_*3*_ and *T*_*4*_ are inoperative and there is no need to estimate them when dealing with leatherback turtles.

**Table 1:** Estimates of the STAMM parameters for juvenile leatherback turtles in the North Atlantic Ocean

Species-dependent parameters	
*v*_0_	1.2 m s^− 1^
*T*_*1*_	*T*_*1*_ = 24–1.05 M^0.5^
*T*_*2*_	*T*_*2*_ = 24–0.21 M^0.5^
*T*_*3*_ *,T*_*4*_	Inoperative and hence not estimated
Parameters depending on simulated ocean properties	
α	3 10^6^
*P*_0_	80 mmol C m^− 2^ day^− 1^

M is the mass of the simulated individual in kilograms

Pivotal temperatures (*T*_*1*_ to *T*_*4*_) are in °C

The last two parameters (α,*P*_0_) depend on the oceanographic characteristics of the area where dispersal occurs. *P*_0_ is the NPP threshold value above which the adults’ foraging habitat suitability is maximum (*h*_*F*_ = 1). GL suggest using a value of *P*_0_ corresponding to the 90th percentile of the NPP distribution in the area of interest. As the ocean productivity is generally higher in the North Atlantic than in the North Pacific Ocean, this 90th-percentile rule leads us to set *P*_0_ = 80 mmol C m^−2^ day^− 1^, a value larger than that used by GL in the Pacific (*P*_0_ = 55 mmol C m^−2^ day^− 1^). Sensitivity experiments (see the sensitivity analysis section below) suggest that this choice is pertinent. Finally, as explained by GL, the value of α (the parameter that controls the dispersion of the swimming direction around its mean) depends on the median value of the simulated habitat gradient ‖**∇*h***‖ in the area of interest. Since the median values of ‖**∇*h***‖ in the North Atlantic and the North Pacific prove to be almost identical, we simply use here the value of α that GL used in the North Pacific. Estimates of all used model parameters are summarized in Table 1.

Final closure of the model requires the specification of the normalized food requirement as a function of age, as well as a mass-RMR relationship, a mass-size relationship and a growth curve. Here again, we use the same expressions as GL, taken from [40–42]:

10 $$ {F}_0(a)={f}_0\ \frac{x\ {\left(1-x\right)}^{1.86}}{1-{\left(1-x\right)}^{0.094}}\kern0.5em \mathrm{with}\kern0.5em x={e}^{-0.299\kern0.5em \left(a+0.17\right)}\kern0.5em \mathrm{and}\kern0.5em {f}_0=0.094 $$

11 $$ RMR\sim {M}^{0.831} $$

12 $$ M=112.31\ {L}^{2.86} $$

13 $$ L(a)=1.43\ \left[1-{\mathrm{e}}^{-0.226\left(a+0.17\right)}\ \right] $$

where *L* is the straight carapace length (SCL) in meters and *M* is in kilograms. The values of the exponents in Eqs. (11) and (12) imply *b* = 0.831 and *c* = 2.86 so that Eq. (6) finally reduces to:

14 $$ {V}_m={v}_0\ {L}^{0.126} $$

This closes the model calibration.

### Simulations of juvenile leatherbacks dispersal in the North Atlantic Ocean

#### Technical setup

The technical setup of our simulations is directly inherited from that of GL. In this case, we release simulated leatherback hatchlings off the FGS coast but use the same data sources as GL for surface currents, NPP and water temperatures. We also use the same trajectory simulation software, the same hatchlings release procedure, the same model parameters (except for *P*_0_ as explained above) and the same model integration period (18 years).

More precisely, surface currents (***V***_***c***_) are taken from daily outputs of the GLORYS-1 (G1) reanalysis of the World Ocean circulation [43] performed by the Mercator-Ocean centre (http://www.mercator-ocean.fr) with the NEMO numerical ocean model (www.nemo-ocean.eu). The G1 model has an eddy-permitting horizontal resolution of 0.25° and 50 vertical layers. It covers a 7-year period going from 01/01/2002 to 31/12/2008.

Thermal habitat suitability index is estimated using the water temperature in the first layer (0 to 1 m) of G1 while *h*_*F*_ is computed using satellite-derived NPP estimates from the Ocean Productivity web site (www.science.oregonstate.edu/ocean.productivity/). These estimates are obtained with the VGPM algorithm [44]. They are available for the whole G1 period with a spatial resolution of 1/6° and a temporal resolution of 8 days. Linear interpolation in time and bilinear interpolation in space is used to estimate daily NPP values at the centre of each G1 grid cell.

Individual trajectories are computed using the ARIANE Lagrangian trajectory simulation software (www.univ-brest.fr/lpo/ariane) with a daily time step and turtle velocities given by Eq. (1). For comparison purposes only, passive drift trajectories are also computed using ARIANE fed with G1 surface currents alone. Trajectories are computed over a period of 18 years which likely covers the whole pelagic juvenile stage. To perform 18-year-long simulations with 7-year-long forcing data sets, we simply loop the forcing fields until the last released turtle reaches the age of 18.

#### Hatchlings release procedure

**Fig. 1:**
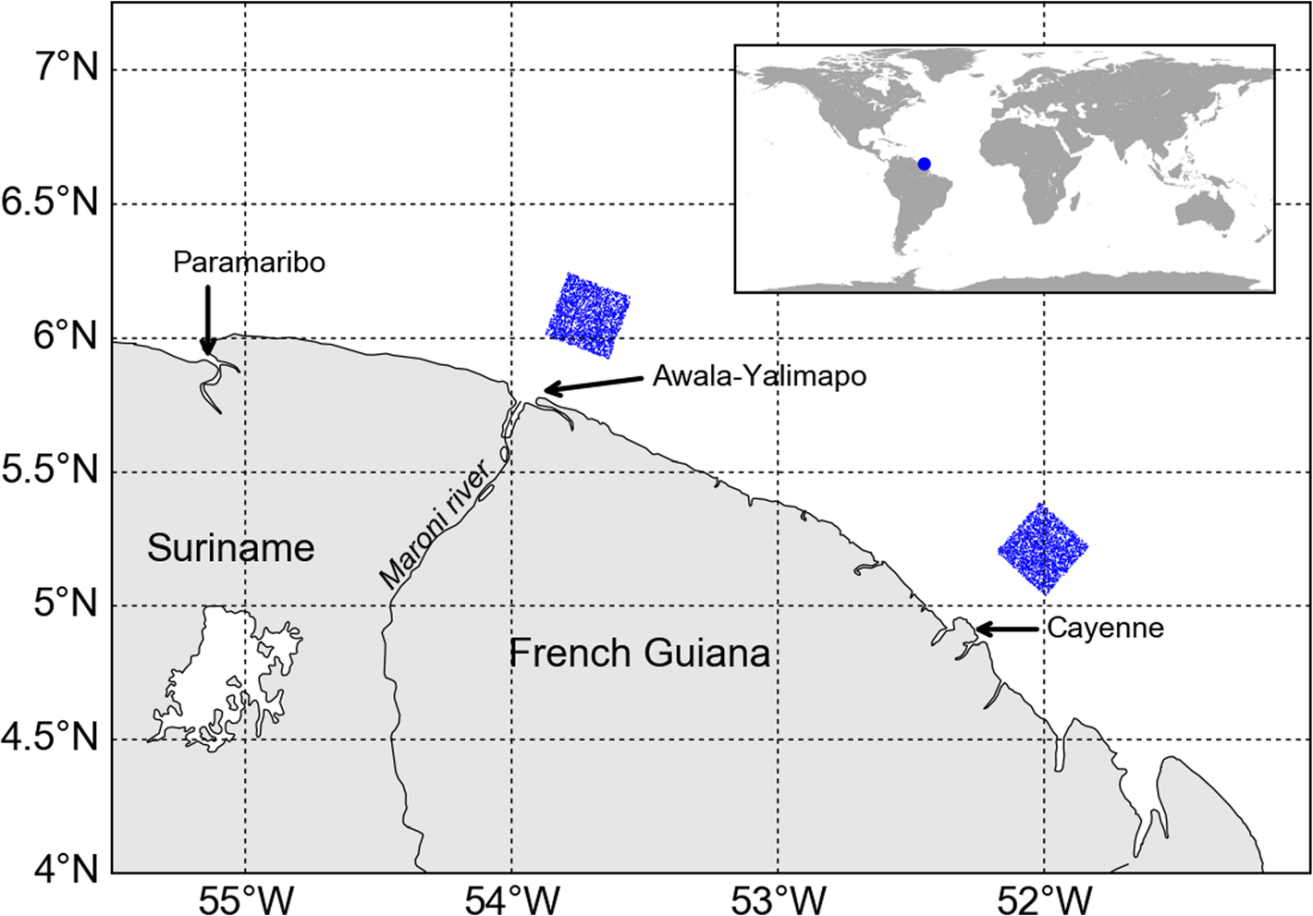
Map of French Guiana/Suriname (FGS) coastline. The blue rectangles represent the hatchling release areas off the Cayenne peninsula and the Maroni estuary

Leatherback nesting along the FGS coast occurs on a number of beaches located between Paramaribo (Suriname) and Cayenne (French Guiana) [2]. This stretch of coastline (Fig. 1) hosts scattered nesting sites of various importance and two main nesting aggregations located on the Cayenne peninsula and near the Maroni estuary, which includes the important nesting beach of Awala-Yalimapo [3]. By the sea, the Maroni estuary lies roughly 200 km northwest of Cayenne and Paramaribo is about 150 km further west. Hatchlings swimming away from any of FGS nesting beaches will rapidly encounter the Guiana coastal current which flows northwestward at a mean speed of 0.4 to 0.5 m/s [45]. At such a speed, it will take 5 to 6 days for hatchlings to drift from the Cayenne area to the Maroni estuary and about 4 more days to reach Paramaribo. Very little difference is thus expected in the timing and shape of the dispersal patterns of hatchlings emerging from any nesting beach between Cayenne and Paramaribo. We will therefore perform a single dispersal simulation in which 5000 hatchlings are released, half of them off Cayenne and the other half off the Maroni estuary. The hatchlings release procedure is the same as that used by GL. The effect of the swimming frenzy is simulated by releasing hatchlings in two 0.25° × 0.25° areas centered about 40 km off the coast (Fig. 1). Release positions are uniformly distributed within these two areas. As the main nesting season extends from mid-March to mid-August and the incubation period lasts about 2 months, hatchlings are released between mid-May and mid-October. The number of releases per day fits a truncated normal distribution that peaks on August 1st.

## Results and discussion

**Fig. 2:**
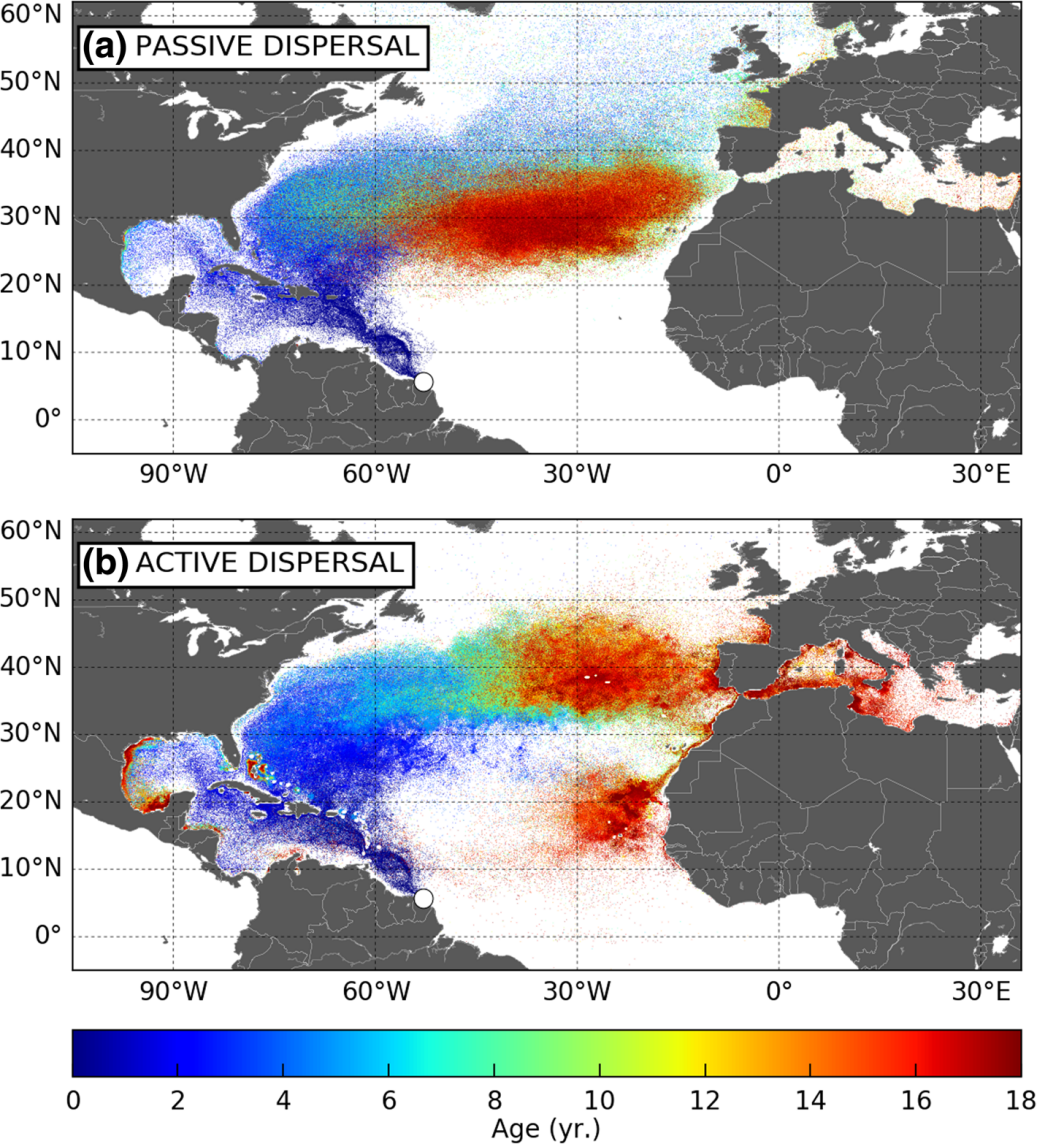
18-year long trajectories of (**a**) passive and (**b**) active juvenile leatherbacks dispersing from their FGS nesting sites. The white dot on the map straddles the two release zones. The color along each track evolves with the age of the simulated turtle

Figure 2 shows the simulated 18-year long trajectories of juveniles (a) passively drifting from their FGS nesting beaches or (b) actively dispersing according to the STAMM model. These two simulations will be referred to as the passive and active dispersal simulations. Accordingly, simulated individuals will be referred to as active and passive turtles. An animated version of the simulated turtle positions evolving with time is provided in Additional file 1 (passive turtles) and Additional file 2 (active turtles).

### Comparison of the active and passive dispersal patterns

#### Initial pathways

**Fig. 3:**
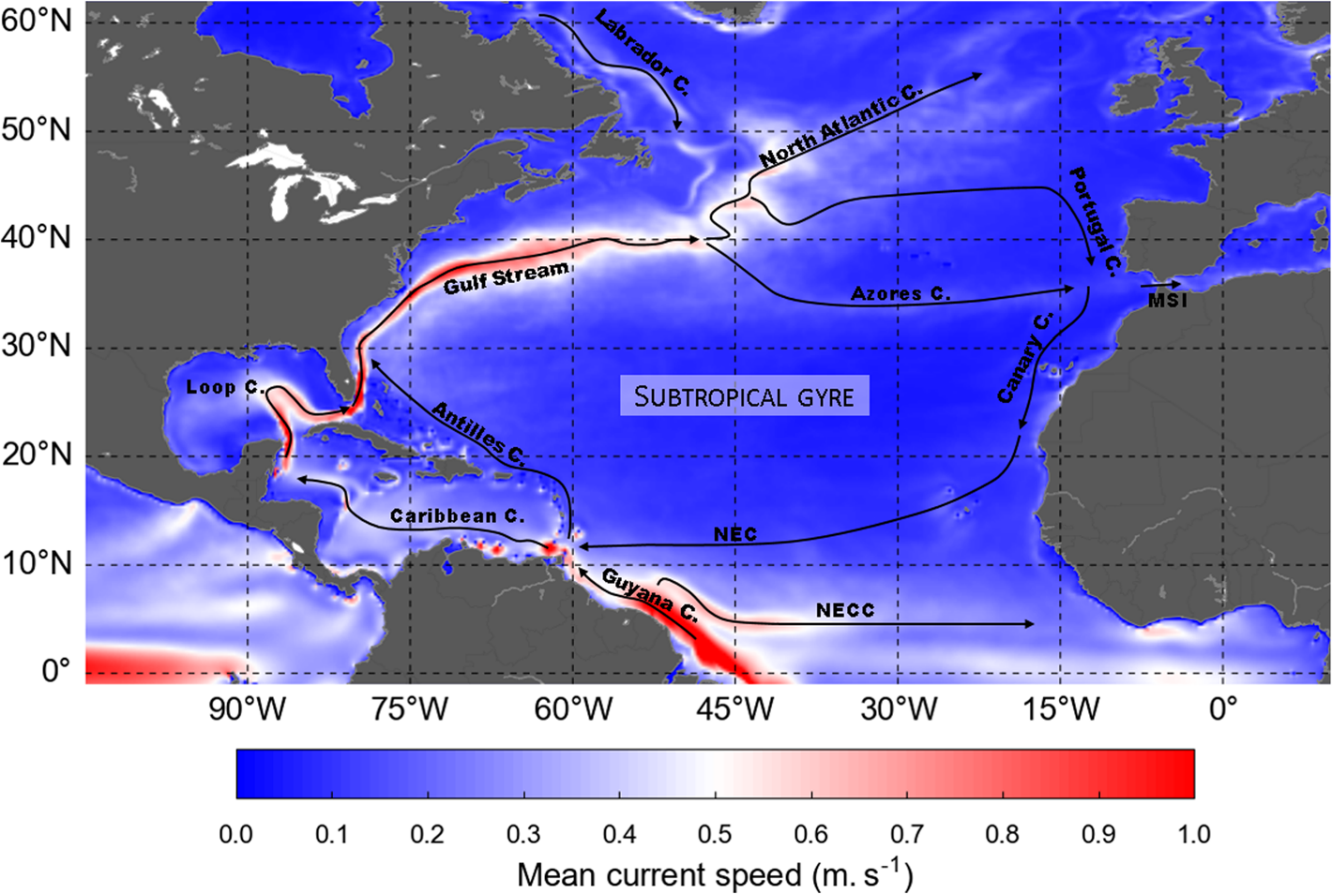
Schematic map of the North Atlantic surface circulation. LC: Loop Current; NAC: North Atlantic Current; NEC: North Equatorial Current; NECC: North Equatorial Counter Current; MSI: Mediterranean Surface Inflow. The coloured background represents the mean current speeds over the whole G1 ocean reanalysis period

As previously observed in GL simulations, large-scale, active and passive, dispersal patterns are broadly similar and mainly shaped by ocean currents (Fig. 3).

Dispersal follows the clockwise circulation around the north Atlantic subtropical gyre. All passive and active hatchlings emerging from the FGS nesting beaches are first entrained northwestward by the mighty Guiana current and rapidly reach the lesser Antilles. From there they keep moving north. Part of them follow the Antilles current east of the Caribbean islands. The others follow the Caribbean current and then the Loop current into the Caribbean Sea and, eventually, the Gulf of Mexico (where some active turtles recruit to rich coastal habitats). Both pathways then converge east of Florida Straits, at about 25°N, a latitude reached by active and passive turtles within about 1 year. During that first year of life, most turtles circulate in warm waters and have modest, thus easily met, food requirements. Accordingly, their habitat suitability index is generally equal or close to 1 so that the swimming velocity of active turtles remains close to zero. This is the reason why active and passive turtles follow nearly identical, essentially passive, dispersal routes. Differences appear later when turtles continue their clockwise journey, progressively veering north then east to cross the Atlantic.

#### Crossing of the North Atlantic Ocean

**Fig. 4:**
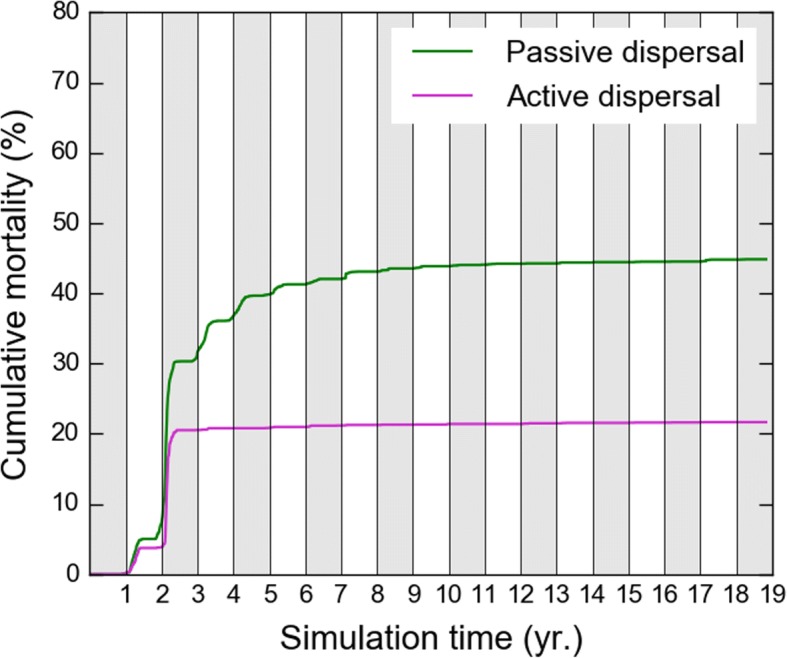
Cumulative cold-induced mortality in passive and active turtles

This second part of the journey is more dangerous as turtles now navigate at higher latitudes where they can encounter water temperatures well below the critical temperature *T*_*1*_. Assuming, like GL, that death occurs when a turtle experiences *T*_*w*_ < *T*_*1*_ during at least 10 days, our simulations show (Fig. 4) that cold-induced mortality is maximum, in both active and passive turtles, during the second winter at sea. At the end of it, cumulated cold-induced mortality reaches 20,5% in active turtles and 30,3% in passive turtles. Mortality is essentially observed amongst the individuals that are rapidly entrained northward by the Gulf Stream. It is less frequent in the individuals which circulate at lower latitudes inside the subtropical gyre. Reduced mortality in active turtles obviously indicate that their swimming activity, although still limited, is already sufficient to help them escape overly cold waters in some (but not all) cases.

After that second winter at sea, cold-induced mortality becomes very small in active turtles, indicating that they are then sufficiently cold-resistant and powerful swimmers to retreat fast enough towards warmer waters (that is generally southward) to avoid cold-stunning during fall and winter. During spring and summer, they migrate back north as the water warms up and gives them access to more productive areas found at higher latitudes. While performing these north-south seasonal migrations, active turtles progressively move east under the influence of the Gulf Stream and then the North Atlantic or the Azores Current (see animation in Additional file 2). Under the influence of the same eastward currents, passive turtles also progressively cross the Atlantic. They do not migrate seasonally but disperse over a wide range of latitudes, sometimes well above 50°N (Fig. 2a). As a consequence, their cumulated cold-induced mortality reaches 45% at the end of the simulation. In the rest of this paper, turtles diagnosed as dead by cold will be discarded from the analysis.

#### Final dispersal

Towards the end of the simulation, most passive turtles end up accumulating in the centre of the subtropical gyre, just like plastic debris [46]. This oligotrophic area is unlikely to be a suitable development ground. Coupled with the high cold-induced mortality rate, this observation suggests that passive dispersal is not a realistic hypothesis for juvenile leatherbacks crossing the North Atlantic.

The final fate of active turtles is markedly different. Thermal constraints keep them generally well below 50°N all year through, down to 35°N or below during wintertime (see Fig. 2b and Additional file 2). Most of them perform seasonal migrations every year, keep progressing eastwards with the dominant currents and finally arrive off the coast of Europe or Northern Africa, typically between 30°N and 45°N. Summer and fall arrivals take place in the northern half of this latitude range between the Bay of Biscay, the coast of Galicia and the northern coast of Portugal. Individuals arriving during winter and spring land further south, between the southern coast of Portugal and Morocco. These are generally productive areas where simulated swimming speeds are weak. Under the influence of the Portugal current, most of these turtles are progressively entrained southwards. On the way, some of them get attracted into the rich Cadiz Bay where productivity is enhanced by the Guadiana and Guadalquivir river outflows [47]. These turtles are then entrained by the Mediterranean surface inflow, pass through the Strait of Gibraltar and finally disperse into the Mediterranean Sea.

The rest of the turtles keep moving south with the Portugal and then the Canary current. Following the Moroccan coast, they finally reach the Mauritanian upwelling area where most of them remain until the end of the simulation. Interestingly, a few (< 1%) active turtles manage to arrive much earlier in this area. Often after a southward wintertime migration, these individuals happen to find sufficiently favorable feeding habitats inside the subtropical gyre and do not migrate back to higher latitudes. Being pushed by generally eastward currents they arrive off Mauritania within about 4 years. Their (blue) trajectories are clearly visible in the [20–30°N; 50–30°W] area in Fig. 2(b). They are the very first NWA juveniles to complete their crossing of the North Atlantic basin, albeit at relatively low latitudes (mostly between 20 and 30°N).

This complete dispersal scenario comes with a rather low cold-induced mortality rate (21.7% at the end of the simulation, Fig. 4) and leads simulated juveniles towards various favorable developmental areas along the coast of Europe and Northern Africa. It thus seems more likely than the passive drift scenario but can it be further corroborated by observations?

### Matching simulated dispersal scenarios with observations

Observations concerning juvenile leatherbacks dispersal in the North Atlantic (or anywhere else) are very few. In the absence of electronic tracking data and conventional tags returns, bycatch and stranding data constitute our main source of information. Unfortunately, quantitative use of such data is difficult. In particular, they can hardly be used to estimate abundances at sea, or even abundance variations, that could be compared to simulated values. Indeed, the observation effort (and thus the detection probability) associated with presently available stranding or bycatch data sets is rarely quantified. In addition, this observation effort varies among the different observation regions and with time (specially in data sets spanning several decades, see Table 2). Furthermore, the local mortality, which links the number of stranded or bycaught turtles to the local abundance of turtles at sea, is typically unknown and varies with the age-class, the region and the period of observation (e.g. [47]). The transfer function between local abundances and observed strandings or bycatches is thus presently unidentifiable at the population level and, *a fortiori,* at the age-class level.

**Table 2:** Synthesis of leatherback bycatch and stranding data gathered in various sites along the Atlantic coast of Europe and North Africa, and in the Mediterranean Sea

Area	Observed individuals	Measured individuals	Period of observation	CCL (in cm)				Source
				Min	Max	Mean	STD	
Mauritania	183	48	2008–2011	62	187	118.3	34.6	Coelho et al., 2015 [51]
Portugal	337	187	1978–2013	80	200	139	18.5	Nicolau et al., 2016 [47]
Gulf of Cadiz	102	30	1960–1996	100	195	136.5	17.6	Caminas and Gonzalez de la Vega, 1997 [53]
Tunisia	51	35	1907–2011	100	210	158	29.2	Karaa et al., 2013 [54]
Bay of Biscay	391	272	1995–2015	102	210	147.9	16.9	Dell’Amico, 2017 (Pers.com)
Galicia	241	87	1849-2013	93	192	146.1	20.0	Lopez et al.,2014 [56]

Nevertheless, when individual sizes are recorded, stranding or bycatch observations can confirm the presence of juveniles in different parts of the Atlantic basin and, assuming a growth curve, can give an indication of the timing of the dispersal events that led these individuals from their nesting beaches to the place where they were observed.

In theory, the origin of the observed individuals should be ascertained by genetic analyses. In practice, this is rarely the case. Luckily however, there is little doubt that leatherbacks encountered in the North Atlantic belong to the NWA subpopulation. Indeed, multiple tracking studies [6, 7, 9] and genetic analyses [48, 49] show that a clear spatial separation exists between the NWA subpopulation and the other two Atlantic subpopulations: the very small South West Atlantic subpopulation nesting in Brazil and the much larger South East Atlantic subpopulation nesting mostly in Gabon. While the NWA subpopulation is widely present in the North Atlantic, the African and Brazilian subpopulations appear to be confined within the southern hemisphere. One can thus reasonably assume that all leatherback bycatch and stranding data in the North Atlantic concern individuals from the NWA subpopulation.

#### Observed and simulated hotspots

**Fig. 5:**
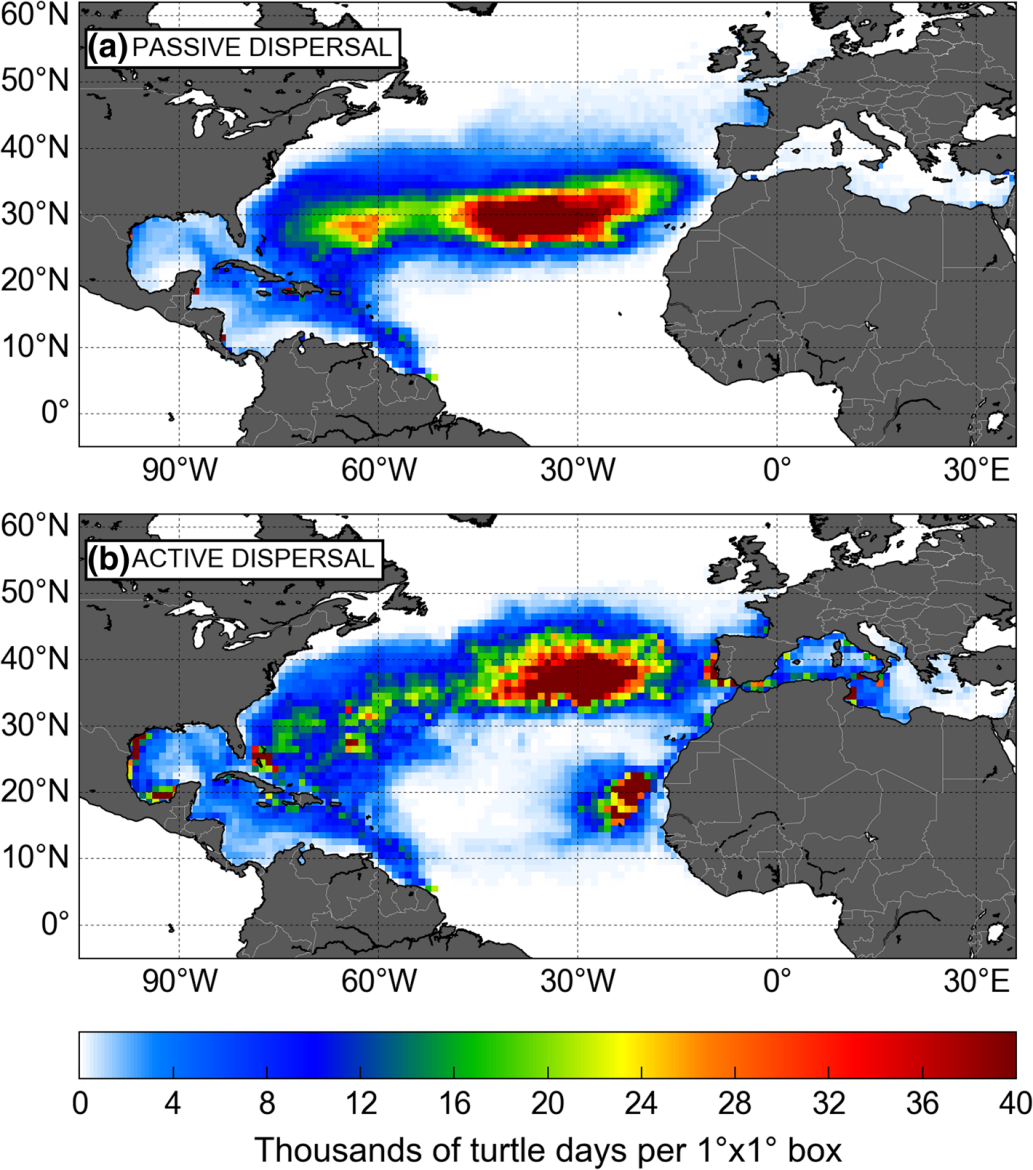
Number of turtle days recorded in 1°× 1° boxes during the (**a**) passive and (**b**) active dispersal simulations. Unlike in Fig. 2, turtles having suffered cold-induced mortality are not taken into account

The validation of simulated dispersal patterns requires that the presence of juveniles be confirmed, at least, in the areas most frequently visited by simulated turtles. These hotspots are easily identified in maps showing the number of simulated daily positions (or “turtle days”) recorded in regular boxes of, say, 1° × 1° (Fig. 5). As expected, differences in the spatial distributions of active and passive turtles are most evident in the central and eastern parts of the basin where active individuals are older/bigger and thus have a more significant swimming activity.

The distribution of active turtles displays two main hotspots: the first one around the Azores (mostly within the archipelago and north of it) and the second one off Mauritania (this area also includes the Cape Verde Islands). A smaller, but well-marked, coastal hotspot is present along the Portuguese coast, extending into the Bay of Cadiz. Somewhat lower, but still significant, turtle densities are observed in the Bay of Biscay and along the Galician and the Moroccan coast. Active turtles are also present in the Mediterranean Sea, essentially in the western basin where some small coastal hotspots are visible. The main one is observed along the east coast of Tunisia. On the contrary, passive turtles concentrate only in the center of the subtropical gyre, south of the Azores archipelago. Their density is very low along the French, Spanish, Portuguese and Moroccan coast. They do no visit the Mauritania/Cape Verde area and very few of them (< 0.8%) manage to enter the Mediterranean Sea.

A rapid search of the literature confirms the presence of stranded or bycaught leatherbacks in all main hotspots identified in the active dispersal simulation: in the Azores area [50], off Mauritania [51, 52], along the Portuguese coast [47], in the Gulf of Cadiz [53] and along the Tunisian coast [54]. Stranded or bycaught leatherbacks are also reported in the Bay of Biscay [55], along the Galician coast [56], the Moroccan coast [57], and in the Mediterranean sea [58]. Interestingly, data indicate that leatherback stranding density is markedly higher along the Portuguese coast than in the bay of Biscay or Galicia [47]. It is also higher in the western than in the eastern part of the Mediterranean Sea [58]. These variations in stranding densities are consistent with the simulated spatial distribution of turtle densities in these areas.

These bycatch and stranding observations however include individuals of all sizes. If we conservatively assume that juvenile leatherbacks have a maximum curved carapace length (CCL) of 105 cm [59], individual size records confirm the presence of juveniles in all above-mentioned areas except, quite surprisingly, the Azores. As juvenile leatherbacks are incidentally captured west of the Azores by the US longline fleet [60] and found stranded at different places along the European and north African coastline, it is indeed difficult to imagine how these individuals can cross the North Atlantic Ocean without transiting through or near the Azores. The deployment of electronic tags on juvenile leatherbacks incidentally caught off the US East coast will probably be required to solve that puzzle.

Except in the Azores area where evidence is missing, our simulated active dispersal pattern thus appears to be largely corroborated by extant stranding and bycatch data while the passive dispersal scenario is clearly dismissed.

#### Timing of dispersal events

The size of the smallest juvenile recorded in a given area is particularly informative as it provides an indication of the age of the youngest individuals present in this area. It shall thus be comparable to the age of the youngest simulated turtle entering this area.

Interestingly, bycatch and stranding data gathered in various sites along the European/North African Atlantic coastline and in the Mediterranean Sea (Table 2, more details in Additional file 3) clearly demonstrate early arrival of juvenile leatherbacks off Mauritania. The smallest individual observed there has a CCL of 62 cm, well below the sizes of the smallest individuals observed everywhere else along the European or North African coastline. This observation is consistent with the active dispersal scenario which features a fast pathway towards Mauritania.

Data also suggest that the next arrivals occur along the Portuguese coast where the minimum observed CCL is 80 cm. First arrivals in all other observation areas appear to occur somewhat later, but in a rather short time interval, as minimum CCLs are quite similar (all between 93 and 102 cm).

**Fig. 6:**
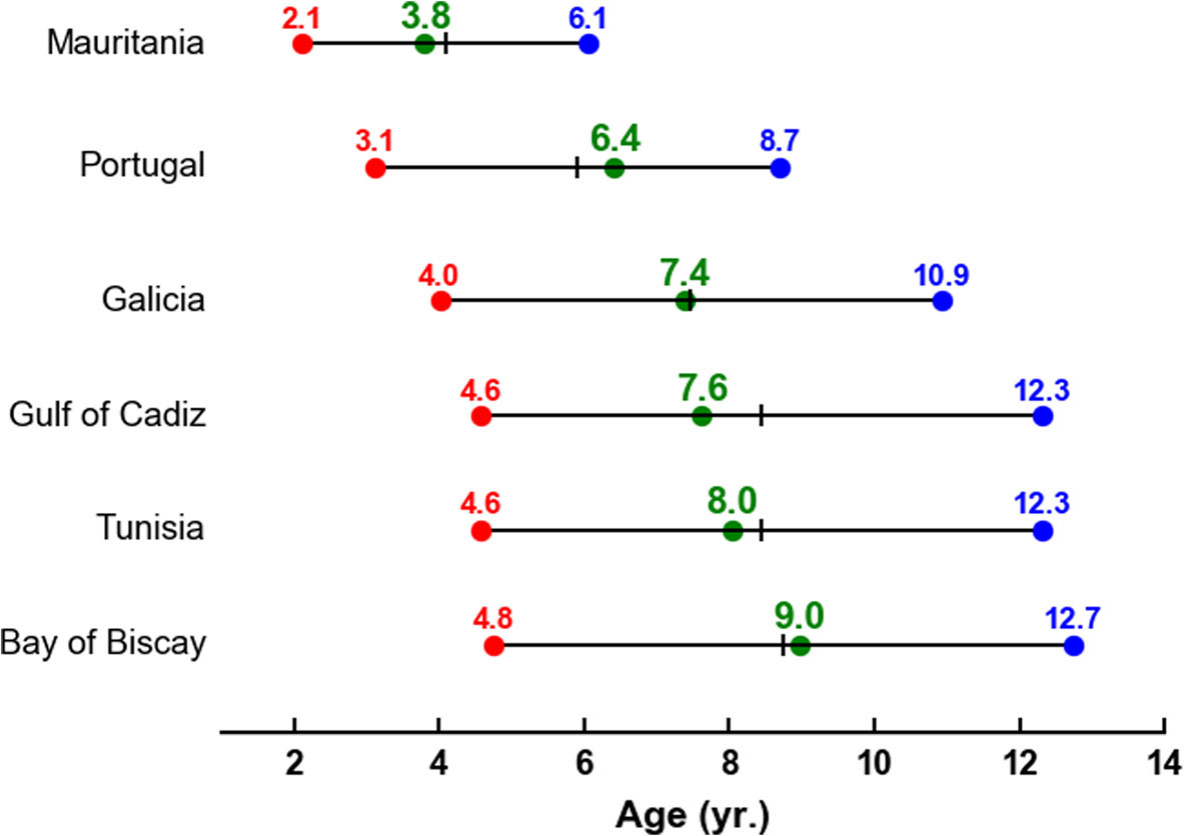
Lower (red dots) and upper (blue dots) age estimates for the smallest individuals observed in different areas, compared with the ages of the simulated active turtles (green dots) first entering these areas. Upper and lower age estimates are derived from the growth curves of Avens et al. [62] and Jones et al. [41] respectively. The midpoint of each estimated age range is indicated by a black tick mark

**Fig. 7:**
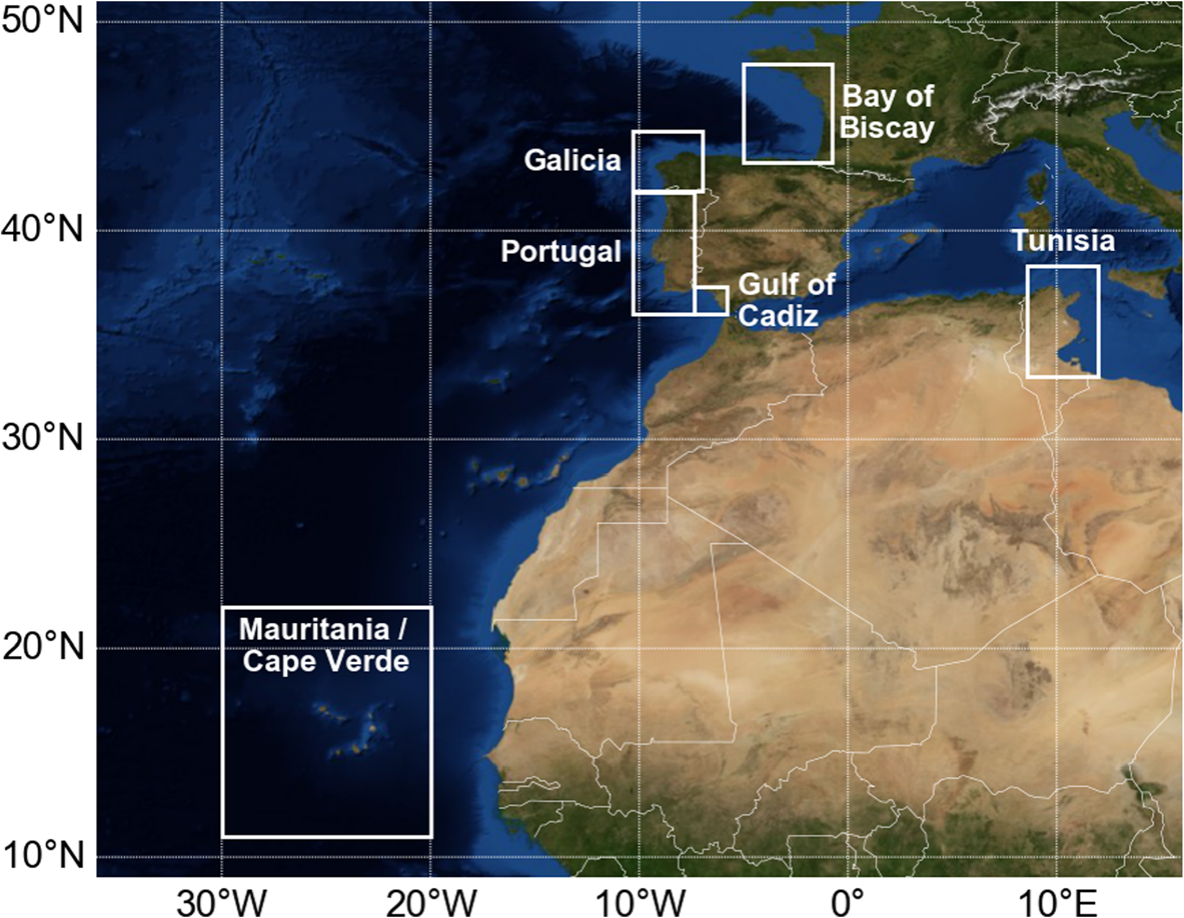
Arrival areas used to determine the age at which active turtles reach the different zones where stranding/bycatch data are available

The likely sequence of arrivals is thus clear but the precise timing of these arrivals is difficult to establish. Indeed, individual growth rates are highly variables in sea turtles [61] so that age estimates based on sizes are specially uncertain. Upper and lower age estimates can however be obtained using, respectively, a slow [62] and a fast [41] growth curve. Using these two curves, upper and lower age estimates are readily obtained for the smallest individuals observed in the different areas listed in Table 2. These can then be compared with the ages at which the youngest simulated turtles arrive in these areas (Fig. 6). The arrival areas used for these computations are shown in Fig. 7. They typically extend 150 km off the coastal areas where strandings are reported. The arrival area off Mauritania [20–30°W; 11–22°N] corresponds to the zone where leatherback bycatches were reported [51] although the vast majority of bycatches actually occurred west of 25°W.

Interestingly, the simulated ages at arrival always fall within the corresponding estimated age range. They actually fall very close to its midpoint (that is the mean age estimate). Furthermore, the simulated order of arrival is consistent with the observations. The first active turtles arriving on the eastern side of the Atlantic basin are observed off Mauritania. They are about 4-year-old. The first arrivals off Portugal happen about 2.5 years later. The first simulated individuals arriving off the coast of Galicia and the Gulf of Cadiz are close to 7.5-year-old and those reaching the Tunisian coast are only a few months older. The first arrivals within the colder Bay of Biscay concern 9-year-old simulated turtles.

The simulated timing of juvenile arrivals on the Eastern side of the Atlantic Ocean is thus fully consistent with the estimated age ranges of the smallest individuals stranded or bycaught along the European and north African coastline.

However, this simulated timing is established for individuals born on the FGS coast while the FGS nesting aggregation accounts for only half of the NWA leatherback subpopulation. The other half nests in the Caribbean area up to Florida, that is well downstream of the FGS nesting beaches. Our simulations indicate that FGS juveniles take about 1 year to arrive off Florida. One can thus reason that individuals born in Florida shall reach the eastern side of the Atlantic up to 1 year faster than those born in FGS. Figure 6 clearly shows that even if these juveniles were arriving 1 year earlier than the FGS juveniles, their arrivals in the different observation areas would still occur within the correct estimated age ranges. Our simulation results are thus consistent with the observations regardless of the exact nesting area from where the juveniles originate.

A last point to note is that active turtles cross the North Atlantic Ocean relatively slowly. This is mainly because their simulated mean zonal swimming velocity, though weak (≈1 cm/s), is westward and thus opposite to the main current direction. Like in the Pacific [31], this simulated westward swimming velocity is due the fact that habitats are, on average, more favorable in the productive and warm western part of the basin than in its central basin. The average habitat gradient thus points westward and so does the average simulated velocity. If simulated turtles did not swim slightly westward, arrivals on the eastern side of the Atlantic (except the early arrivals off Mauritania), would occur about 3 years earlier. Figure 6 indicates that such a faster crossing scenario would still be marginally compatible with stranding data but only if juveniles were growing fast during their whole journey across the Atlantic. While fast growth is likely during the crossing of the relatively warm and productive western Atlantic basin, it is unlikely during the crossing of the oligotrophic central basin, specially during winter. A slight mean westward swimming velocity, as simulated there, thus appears to be needed to explain the sizes of the smallest individuals observed on the Eastern side of the Atlantic Ocean.

### Sensitivity analysis

As pointed out by GL, estimation of the *P*_0_ parameter is the most uncertain part of the model calibration. The value of *P*_0_ used so far is 80 mmol C m^− 2^ day^− 1^. It corresponds to the 90th percentile of the NPP distribution in the North Pacific. With this choice, *h*_*F*_ ≈ 1 in the most productive pelagic areas (generally fronts and eddies) where sea turtles (and many other top predators) are known to forage [63, 64]. To test the sensitivity of STAMM results to this parameter choice we performed three new 18-year-long active dispersal simulations with *P*_0_ equal to 40, 60 and 100 mmol C m^− 2^ day^− 1^, respectively. Higher NPP values were not tested as such values are hardly encountered in the open ocean.

**Fig. 8:**
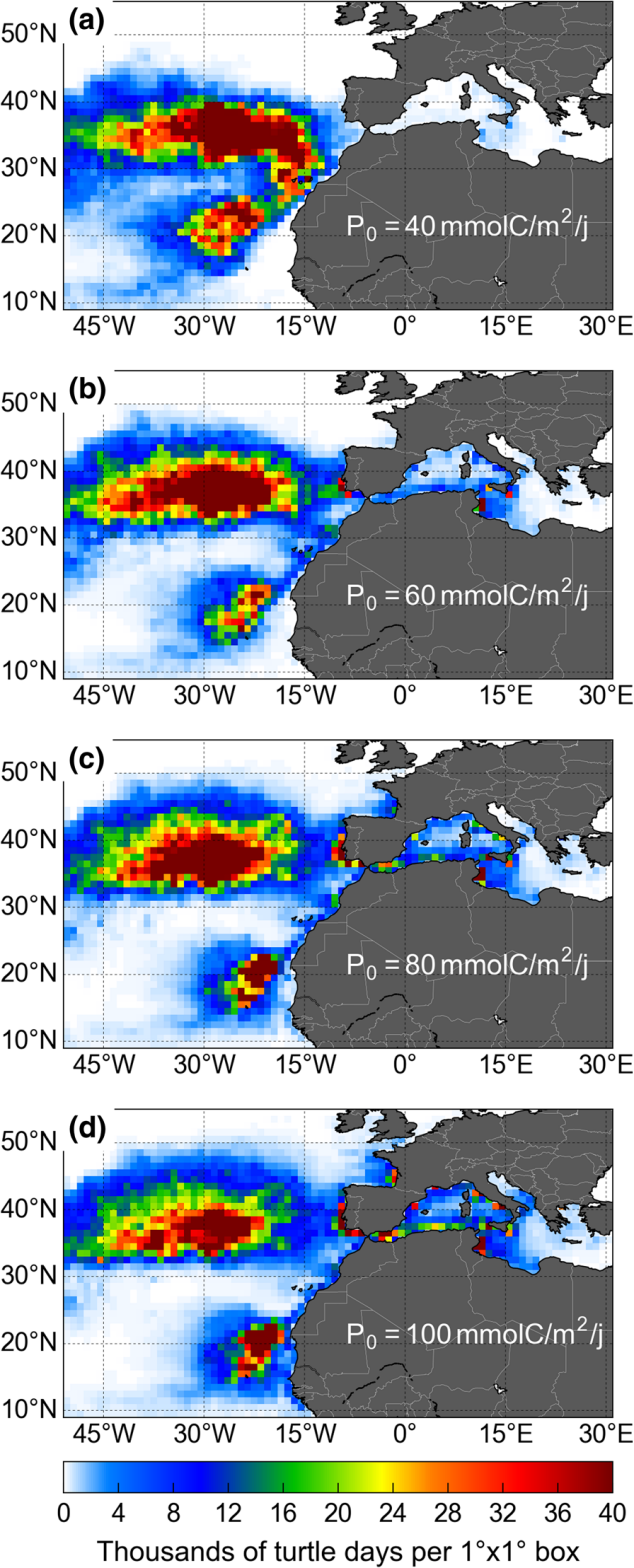
Simulated densities of active turtles in the central and eastern part of the North Atlantic basin for 4 values of the *P*_0_ parameter shown in panels (**a**) to (**d**). Panel (**c**) corresponds to the nominal active simulation (*P*_0_ = 80 mmol C m^− 2^ day^− 1^). Turtles having suffered cold-induced mortality are not taken into account

Setting *P*_0_ = 40 mmol C m^− 2^ day^− 1^ significantly affects the simulated turtle distribution, in particular in the eastern part of the Atlantic basin (Fig. 8a). With such a low *P*_0_ value, *h*_*F*_=1 almost everywhere so that very productive zones are not more attractive than moderately productive ones. Swimming movements are to essentially governed by thermal habitat favorability. Accordingly, simulated turtles are no longer attracted towards the productive but relatively cold Bay of Biscay and Portuguese coast. Very few of them enter the Mediterranean Sea and the Mauritanian hotspot is found further offshore, away from the coldest (but more productive) upwelling area.

The Portuguese hotspot appears only when the value of *P*_0_ is increased to 60 mmol C m^− 2^ day^− 1^ (Fig. 8b). This also causes more turtles to enter the Mediterranean Sea and concentrate along the coast of Tunisia. The Mauritanian hotspot gets closer to the coast where productivity is higher, but turtle densities remain low in the Bay of Biscay. Pushing *P*_0_ up to 80 mmol C m^− 2^ day^− 1^ (the nominal value, Fig. 8c) is needed to attract more turtles into the Bay of Biscay and the Bay of Cadiz. More turtles also enter the Mediterranean Sea and the Mauritanian hotspots is drawn closer to the coast. Note that increasing *P*_0_ favors entrance into the Mediterranean Sea not because it makes it more attractive (Mediterranean NPP values are not specially high) but because it makes the productive Gulf of Cadiz more attractive compared to the neighboring areas. Simulated movements are thus more oriented towards this Gulf. Once there, active turtles are naturally entrained into the Mediterranean Sea by the surface inflow.

Further increasing *P*_0_ to 100 mmol C m^− 2^ day^− 1^ causes the Mauritanian hotspot to move further inshore thereby causing the simulated turtles densities to diminish west of 25° W while, on the contrary, observations indicate that bycatch rates increase in that area [51]. The simulated turtle density in the Bay of Biscay also increases and becomes comparable to the density off the Portuguese coast, in contradiction with observed stranding densities [47].

This sensitivity analysis thus reveals that the nominal choice of *P*_0_ = 80 mmol C m^− 2^ day^− 1^ is appropriate. Increasing or decreasing its value by 20 mmol C m^− 2^ day^− 1^ or more appears to deteriorate the match between the simulated dispersal patterns and observations.

### Comparing dispersal conditions in the North Atlantic and North Pacific Oceans

During the last few decades, the West Pacific and NWA leatherback subpopulations have followed very different demographic trends. The West Pacific subpopulation has declined 83% during the last three generations and is considered critically endangered [65]. In the same time, the NWA subpopulation has increased by about 20% [1]. The causes of these opposite trends have not been elucidated so far.

Explanations of demographic trends in sea turtle populations are often sought in environmental or anthropogenic factors affecting either nests or adults [66–73]. Pelagic juveniles are likely impacted by factors similar to those impacting adults at sea (prey availability, incidental catches, pollution, etc.) but the juvenile stage is so cryptic that this has rarely been studied so far. Comparison of our active dispersal simulation results with those of GL in the West Pacific is thus of special interest as it allows us to investigate, for the first time, whether environmental conditions encountered during the juvenile pelagic phase can be part of the explanation why leatherbacks are thriving in the Atlantic and declining in the West Pacific.

The first point to note is that the simulated cold-induced mortalities (over the whole simulated period of 18 years) are similar in both oceans but for different reasons. In the West Pacific, hatchlings born in New Guinea can either drift northward into the Kuroshio and rapidly enter cold mid-latitude waters, or they can drift eastward into the North Equatorial Counter Current (NECC) and stay for several years into tropical waters [31] . Cold-induced mortality is high (up to 45%) in the first case and much lower (< 15%) in the second case. The global mortality rate (19,3%) is an average between these two very different cases. In the West Atlantic, all hatchlings emerging from the FGS nesting beaches circulate northward and most of them drift north of 30°N into the Gulf Stream. However, between 30 and 45°N, average water temperatures in wintertime (when cold-induced mortality is maximum) are about 3 °C higher in the Atlantic than in the Pacific. Accordingly, the juvenile cold-induced mortality remains moderate (20,5%) and almost identical to the Pacific one. Cold-induced mortality is thus unlikely to be among the factors explaining the different demographic trends in the NWA and West Pacific leatherback subpopulations.

**Fig. 9:**
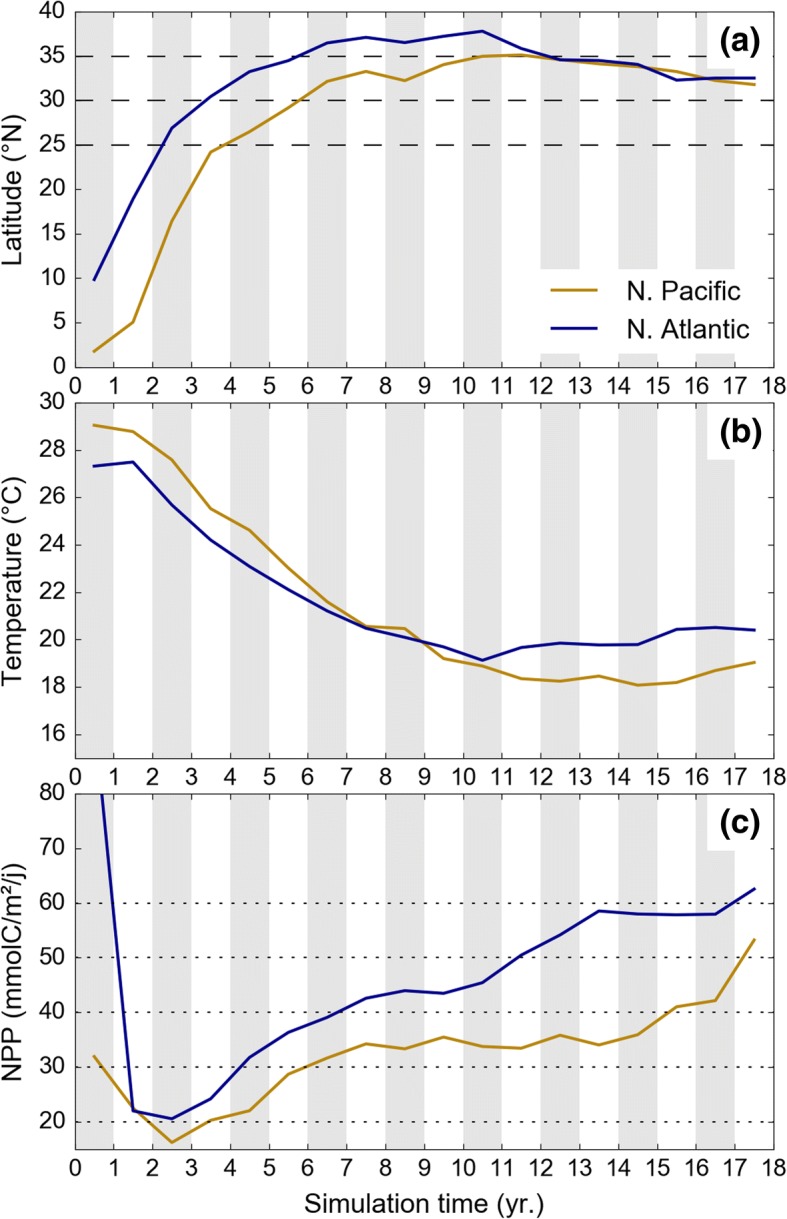
Evolution as a function of time of (**a**) the mean latitudes at which active leatherback turtles cross the North Pacific and North Atlantic basin, (**b**) the mean surface water temperatures and (**c**) the mean NPP encountered by these turtles when crossing the two oceanic basins. Results for the North Pacific are taken from the GL active dispersal simulation

The relatively warm waters found in the North Atlantic not only maintain a relatively low cold-induced mortality but also allow active juveniles to reach higher latitudes during their crossing of the Atlantic. Figure 9a indeed shows that active turtles disperse about 5° further north in the Atlantic than in the Pacific but encounter similar water temperatures (Fig. 9b). They do so to access more productive areas in an ocean that is already, on average, richer than the North Pacific. During the first 10 simulated years, average NPP values encountered by active turtles crossing the Atlantic are thus 5 to 15 mmol C m^− 2^ day^− 1^ larger than those encountered by Pacific juveniles (Fig. 9c). This difference increases markedly after year 10, quickly reaching 40 mmol C m^− 2^ day^− 1^ or more, as, by then, most Atlantic turtles have reached the productive areas off the western coast of Europe and North Africa. The NPP encountered by Pacific turtles increases much later (at the beginning of year 15) when they enter the rich California current region. In both oceans, arrival in the rich eastern boundaries areas likely signals the beginning of a period of rapid energy accumulation after which the first reproductive migration might occur. This implies that Atlantic juveniles not only benefit from better foraging habitats during their whole pelagic phase but also likely reach sexual maturity several (typically 5) years before Pacific juveniles. The Atlantic Ocean being narrower than the Pacific, their return trip towards their nesting beaches is also shorter and thus less energy consuming. These three factors likely contribute to the enhancement of the reproductive output of NWA leatherback turtles and could, at least partly, explain why their subpopulation is doing much better than the West Pacific one.

## Summary and conclusion

This paper presents the first detailed simulation of the spatial and temporal distribution of juvenile leatherback turtles dispersing from their FGS nesting beaches into the North Atlantic Ocean and the Mediterranean Sea. This 18-year-long simulation is performed using STAMM, an IBM in which juvenile sea turtles actively disperse under the combined effects of oceanic currents and habitat-driven swimming movements.

Simulation results suggest that, while a few juveniles cross the north Atlantic basin at relatively low latitudes and arrive off Mauritania within 4 years, most active turtles cross this basin at mid-latitudes while undertaking important north-south seasonal migrations. After several years, they reach the European or north African coast. Some of them cross the Strait of Gibraltar and disperse essentially in the western Mediterranean Sea. Arrival of slightly less than 7-year-old individuals is simulated off the rich Portuguese coast. Other productive coastal areas (the coast of Galicia, the Gulf of Cadiz, the Tunisian coast and finally the Bay of Biscay) are reached by 7 to 9-year-old turtles. Part of these turtles are entrained further south along the north African coast by the Portugal and then the Canary currents. They finally also reach the Mauritanian upwelling area.

This simulated active dispersal scenario appears to be consistent with all stranding and bycatch data gathered along the coasts of Europe or North Africa, and in the Mediterranean Sea. It is spatially consistent as the hotspots identified in the active simulation correspond to areas where juvenile strandings or bycatches have actually been reported. It is also temporally consistent as the ages of the first individuals reaching different hotspots are consistent with the estimated ages of the smallest bycaught or stranded individuals reported in these areas.

The inclusion of simulation of active movements appears to be indispensable to reach this level of consistency with observations. This is specially clear in three cases:

1. Observations of very small individuals off Mauritania [51] tend to confirm the existence of a fast pathway to this area. Such a fast pathway is completely absent from passive simulations and appears to exist only because southward wintertime migrations can occasionally lead juveniles into the subtropical gyre, along a direct pathway towards Mauritania.
2. Entrance in the Mediterranean Sea is another dispersal pattern that essentially depends on the existence of active movements. Sensitivity experiments indeed show that active turtles must first actively swim towards the Gulf of Cadiz (where they are attracted by highly productive waters) before flowing through the Strait of Gibraltar with the Mediterranean surface inflow.
3. Analysis of the simulated swimming velocities suggests that, like in the North Pacific, juveniles crossing the North Atlantic tend to occasionally swim against the dominant eastward currents. This delays their arrival on the eastern side of the Atlantic basin by about 3 years. Without these 3 years, the ages of the simulated turtles would hardly match with the estimated ages of the individuals bycaught or stranded along the coast of Europe and North Africa.

While an enigma remains about the presence of juvenile leatherbacks in the Azores area, our simulations results corroborated by stranding and bycatch data, suggest that the rich Portuguese coastline, the Bay of Cadiz and the Mauritanian upwelling area are major hotspots exploited by NWA juvenile leatherbacks. The coast of Galicia and, further away, the Bay of Biscay also appear to be exploited, although less extensively. Within the Mediterranean Sea, the Tunisian coast is another important foraging area. Hopefully, our results will help focus observation and conservation efforts in these critical zones.

The comparison or our simulation results with those obtained by GL in the North Pacific suggest that the timing of the dispersal and the quality of the habitats encountered by NWA juveniles can, at least partly, explain why the NWA leatherback subpopulation is doing much better than the West Pacific one.

Finally, even if these results are particularly encouraging and prompt us to continue work with STAMM, it is important to realize that only indirect validation of this model has been obtained so far. We thus recommend that new tracking experiments focusing on juvenile leatherbacks be designed and funded.

## Additional files

Additional file 1: Animated 18-year-long dispersal of passive leatherbacks in the North Atlantic Ocean. Five thousand simulated individuals are released offshore FGS (white dot on the map). Their positions (blue dots) are displayed at 10-days intervals. Dots turn black when cold-induced mortality occurs. Blacks dots disappear after 3 months. (AVI 2910 kb)

Additional file 2: Animated 18-year-long dispersal of active leatherbacks in the North Atlantic Ocean. Five thousand simulated individuals are released offshore FGS (white dot on the map). Their positions (blue dots) are displayed at 10-days intervals. Dots turn black when cold-induced mortality occurs. Blacks dots disappear after 3 months. The coloured background represents the value of the habitat suitability index (*h*). (AVI 3790 kb)

Additional file 3: Details of size data processing for the different bycatch and stranding data sets listed in Table 2. (DOCX 55 kb)

## Abbreviations

CCL: Curved carapace length
FGS: French Guiana and Suriname
G1: GLORYS-1 reanalysis of the World Ocean circulation
GL: Gaspar and Lalire [31]
IBM: Individual Based Model
NWA: Northwest Atlantic
SCL: Straight carapace length
STAMM: Sea Turtle Active Movement Model
