# Supplementary material for: Modeling the active dispersal of juvenile leatherback turtles in the North Atlantic Ocean
Source: Mov Ecol. 2019 Feb 28;7:7. doi: 10.1186/s40462-019-0149-5 (PMC6394021; doi:10.1186/s40462-019-0149-5)
Supplement: Supplementary file 3 — Details of size data processing for the different bycatch and stranding data sets listed in Table 2. (DOCX 55 kb) [file 40462_2019_149_MOESM3_ESM.docx]

**Details of size data processing for the different bycatch and stranding data sets listed in Table 2.**

Stranding and bycatch data sets used to obtain Table 2 are of various origins and contain heterogeneous information. In particular, different size measurements are reported: unspecified carapace length (CL), straight carapace length (SCL), curved carapace length (CCL) or total length (TL). For comparison purposes all size measurements are transformed here into CCL measurements. When SCL measurements are provided, CCL is estimated using the Tucker and Frazer [1] relationship:

CCL = 1.04 SCL + 2.04 (S1)

where both SCL and CCL measurements are in centimeters. When TL is measured, SCL is estimated using the Lopez et al. [2] relationship :

SCL = 0.798 TL (S2)

CCL is then computed using (S1). As suggested in [3], CL measurements are likely to be CCL measurement as these are the easiest to obtain. We thus assume CL = CCL. More precisely, the following processing steps are applied to the different data sets listed in Table 2:

**Mauritania**: Size statistics, as provided in Table 2, are directly retrieved from [4], without any additional processing. Note that this data set only contains longline bycatch data. As indicated by the authors, large individuals were released without being measured. Therefore, the mean and maximum sizes are biased low, but not the minimum size.

**Portugal**: Size statistics, are directly retrieved from [5]. However, the median size and interquartile range (IQR) are reported instead of the mean size and standard deviation (STD). These are estimated assuming that the size distribution is Gaussian so that the mean is equal to the median and STD = 0.74 IQR.

**Gulf of Cadiz**: CL and/or TL measurements are reported in [6]. When both measurements are available, CL is the one used to estimate CCL. Records for which the type of size measurement (CL or LT) is unclear are discarded. Size statistics are then readily computed.

**Tunisia**: Size statistics provided in Table 2 are directly retrieved from [7] without any additional processing.

**Bay of Biscay**: The Bay of Biscay stranding data set was provided by Florence Dell Amico (Pers. com, 2017). CCL and/or SCL measurements are reported. SCL data are used to estimate CCL only when this measurement is missing. Size statistics are then readily obtained.

**Galicia:** TL measurements only are reported in [2] and used to obtain the corresponding CCL estimates. Measurements recorded as “made on incomplete animals” are discarded before computing size statistics.

**REFERENCES**

1. Tucker AD, Frazer NB. Reproductive Variation in Leatherback Turtles, Dermochelys coriacea, at Culebra National Wildlife Refuge, Puerto Rico. Herpetologica. 1991;47:115–24.

2. Lopez A, Covelo P, Valeiras X, Martinez-Cedeira J. Tatarugas marinas nas costas de Galicia, s.XVIII-2013. Eubalaena. 2014;13:2–36.

3. Casale P, Nicolosi P, Freggi D, Turchetto M, Argano R. Leatherback turtles (Dermochelys coriacea) in Italy and in the Mediterranean Basin. Herpetol J. 2003;13:135–9.

4. Coelho R, Santos MN, Fernandez-Carvalho J, Amorim S. Effects of hook and bait in a tropical northeast Atlantic pelagic longline fishery: Part I—Incidental sea turtle bycatch. Fish Res. 2015;164:302–11.

5. Nicolau L, Ferreira M, Santos J, Araújo H, Sequeira M, Vingada J, et al. Sea turtle strandings along the Portuguese mainland coast: spatio-temporal occurrence and main threats. Mar Biol. 2016;163.

6. Camiñas JA, Gonzalez de la Vega JP. Presencia y mortalidad de la tortuga laud (Dermochelys coriacea) en el Golfo de Cadiz (SW de Espana). Cadiz; 1997. p. 14.

7. Karaa S, Jribi I, Bouain A, Girondot M, Bradaie MN. On the occurrence of Leatherback Turtles Dermochelys coriacea (VANDELLI, 1761), in Tunisian waters (Central Mediterranean Sea). Herpetozoa. 2013;26:65–75.
